# Clear cell renal cell carcinoma micrometastasis: Insights from an unconventional cutaneous presentation

**DOI:** 10.1002/ski2.427

**Published:** 2024-07-19

**Authors:** Mohammad Fardos, Heather Kopecky, James Sligh, Jill Browning

**Affiliations:** ^1^ HCA Healthcare/USF Morsani College of Medicine GME‐HCA Florida Largo Hospital Largo Florida USA; ^2^ Department of Dermatology Morsani College of Medicine University of South Florida/Bay Pines VA Healthcare System Bay Pines Florida USA

## Abstract

Clear cell renal cell carcinoma (ccRCC) is a highly lethal cancer known for its propensity to metastasise, yet the mechanisms underlying metastasis are not well defined. Cutaneous metastases from ccRCC are uncommon and typically occur within 3 years post‐nephrectomy, predominantly affecting the scalp, chest or abdomen. Here, we present a unique case of a 75‐year‐old male, previously treated for ccRCC with right radical nephrectomy, who developed a singular skin lesion on the left side of the neck 19 years post‐nephrectomy. The lesion was confirmed as metastatic ccRCC through histopathological analysis, despite negative imaging findings. Micrometastases, characterised by microscopic tumour cell foci in distant sites, pose a significant diagnostic challenge, frequently evading detection on conventional imaging modalities like computed tomography and magnetic resonance imaging. This case contributes to our understanding of ccRCC metastasis, emphasising the necessity for continued clinical vigilance and thorough diagnostic scrutiny, particularly concerning atypical metastatic sites.

## Graphical abstract

We present a unique case of cutaneous metastasis from clear cell renal cell carcinoma (ccRCC), occurring 19 years post‐nephrectomy in a 75‐year‐old male. The case underscores the diagnostic challenge posed by micrometastases and emphasises the importance of continued vigilance for atypical metastatic sites in ccRCC patients.

## 1 INTRODUCTION

Clear cell renal cell carcinoma (ccRCC) is a highly lethal cancer known for its propensity to metastasise. However, the methods through which metastases occur are not well defined. This often leads to metastases appearing in uncommon locations, either at the same time as the primary tumour diagnosis or years after radical nephrectomy.

## 2 CASE REPORT

**FIGURE 1:**
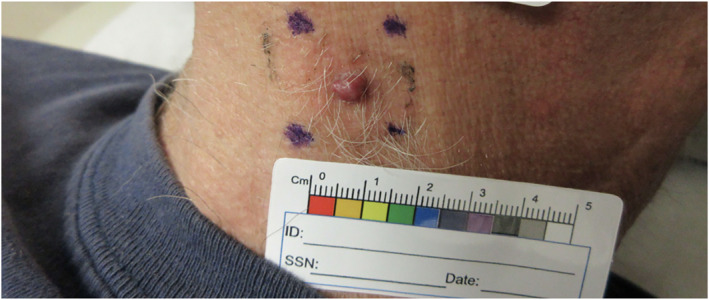
0.7 × 0.5 cm Red to pink coloured papulae overlying a hyperpigmented patch.

**FIGURE 2:**
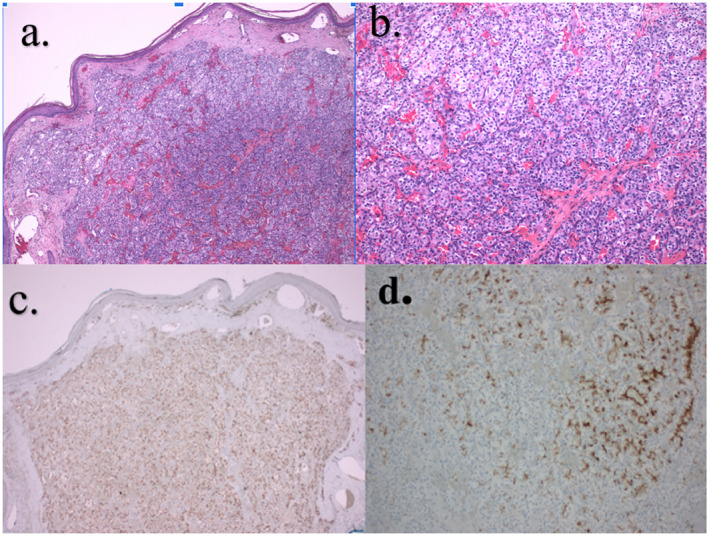
(a, b) Histopathological analysis of the lesion revealed a well‐circumscribed tumour in the dermis composed of nested clear cells with prominent nucleoli intersected by delicate vasculature. (c). Positive PAX8 immunohistochemical stain. (d). Positive RCC immunohistochemical stain.

This was a 75‐year‐old male previously treated for ccRCC with right radical nephrectomy who abruptly developed a singular skin lesion on the left side of the neck 19 years after initial nephrectomy. The original pathology report from radical nephrectomy revealed unifocal grade 2–3 ccRCC with the greatest tumour size of 9 cm extending into the major veins. Additional medical history includes being a former smoker with a 6‐pack year history and having had malignant melanoma 8 years ago, which was treated with wide local excision. There was no reported family history of malignant melanoma, renal cell carcinoma or any other tumours. Immunohistochemical analysis of the melanoma confirmed positive expression for S100, HMB‐45 and Melan‐A. Physical examination revealed a 0.7 × 0.5 cm red‐coloured papulae overlying a hyperpigmented patch (Figure 1). The patient underwent a shave biopsy. Histopathological analysis of the lesion revealed a well‐circumscribed tumour in the dermis composed of nested clear cells with prominent nucleoli intersected by delicate vasculature (Figure 2). Immunohistochemical analysis confirmed the positive expression of pankeratin, PAX8 and RCC in the tumour cells while showing negative results for SOX10, HMB45 and Melan A. A notably elevated proliferative index, as indicated by Ki‐67 labelling, was observed within the tumour. CD10, CK7 and AMCAR immunostaining were not performed in this case. The patient underwent excision of the biopsy‐proven metastatic renal cell carcinoma, which revealed scar/biopsy site changes and no evidence of a residual malignancy. These observations were consistent with a diagnosis of metastatic ccRCC. The patient had a bilateral renal ultrasound performed to evaluate for recurrent renal cell carcinoma, which revealed a prior right nephrectomy and an unremarkable left kidney with several small renal cysts. These findings rule out the presence of bilateral RCCs and suggest that the skin metastasis is not a result of a relapse from a contralateral tumour. The patient had further imaging studies including magnetic resonance imaging of the brain, bone scan and computed tomography of the chest, abdomen and pelvis to evaluate for micrometastasis, which were unrevealing. The patient is being followed by oncology and dermatology for close monitoring and potential consideration for 1 year treatment with Pembrolizumab.

## 3 DISCUSSION

This case reveals the intricate nature of ccRCC metastasis and underscores the importance of heightened clinical vigilance and thorough diagnostic scrutiny, particularly concerning atypical metastatic sites. While ccRCC is known for its predilection to metastasise to distant organs, such as the lungs, liver, bones, lymph nodes, contralateral kidney or adrenal glands; its ability to manifest in unconventional locations adds a layer of complexity to its clinical management.^1^

Patients undergoing curative surgery for ccRCC will often develop metastatic RCC.^2^ Cutaneous metastases from ccRCC are infrequent, constituting only approximately 3% of all skin metastatic tumours.^2^ The timing of skin metastasis detection varies, often occurring within 3 years post‐nephrectomy. However, late‐relapse cases, occurring even a decade after initial surgery have been reported, often being low‐grade.^2^ Cutaneous metastasis presents as a diagnostic conundrum due to their resemblance to more common dermatologic conditions, including haemangiomas, basal cell carcinoma or pyogenic granuloma. Cutaneous metastasis from ccRCC typically occurs in the scalp, chest or abdomen, making the development of cutaneous metastasis in the head and neck region particularly unusual.^1^ Our case, occurring 19 years after nephrectomy in an uncommon location such as the neck, underscores the rarity of cutaneous metastasis from ccRCC and emphasises the importance of continued dermatologic vigilance during long‐term follow‐up.

The optimal treatment for ccRCC remains surgical resection for localised disease, offering the potential for a complete cure with consideration for further immunotherapy.^2^ Micrometastases, characterised by microscopic tumour cell foci in distant sites, pose a significant diagnostic challenge, frequently evading detection on conventional imaging modalities such as computed tomography and magnetic resonance imaging. Flow cytometry of lymph nodes at the time of diagnosis of RCC has been proposed to detect micrometastases in lymph nodes.^3^ In our case, the singular skin lesion prompted further investigation, ultimately confirming metastatic cutaneous RCC through histopathological analysis. This underscores the critical role of histopathology in confirming the diagnosis, especially in cases with negative imaging findings, highlighting the necessity of a comprehensive diagnostic approach.

## CONFLICT OF INTEREST STATEMENT

There are no declared conflicts of interest.

## AUTHOR CONTRIBUTIONS

**Mohammad Fardos**: Data curation (equal); investigation (equal); writing – original draft (lead); writing – review & editing (equal). **Heather Kopecky**: Supervision (equal); writing – original draft (equal); writing – review & editing (equal). **James Sligh**: Supervision (equal); writing – review & editing (equal). **Jill Browning**: Data curation (equal); supervision (equal); writing – review & editing (equal).

## ETHICS STATEMENT

Not applicable.

## PATIENT CONSENT

The authors obtained written consent from patients for their photographs and medical information to be published in print and online and with the understanding that this information may be publicly available. Patient consent forms were not provided to the journal but are retained by the authors.

## Data Availability

Data sharing not applicable to this article as no datasets were generated or analysed during the current study.

## References

[1] Singla A , Sharma U , Makkar A , Masood PF , Goel HK , Sood R , et al. Rare metastatic sites of renal cell carcinoma: a case series. Pan Afr Med J. 2022;42:26. 10.11604/pamj.2022.42.26.33578 35910051 PMC9288148

[2] Mitomi T , Kawahara T , Nomura S , Kuroda S , Takeshima T , Takamoto D , et al. Skin metastasis of renal cell carcinoma. Case Rep Oncol. 2020;13(2):798–801. 10.1159/000508340 32884521 PMC7443632

[3] Hartana CA , Kinn J , Rosenblatt R , Anania S , Alamdari F , Glise H , et al. Detection of micrometastases by flow cytometry in sentinel lymph nodes from patients with renal tumours. Br J Cancer. 2016;115(8):957–966. 10.1038/bjc.2016.279 27599044 PMC5061909

